# Spätmanifestation eines epibulbären ossären Choristoms – 2 Fallberichte

**DOI:** 10.1007/s00347-021-01354-z

**Published:** 2021-03-09

**Authors:** Louisa Bulirsch, Martina C. Herwig-Carl, Frank G. Holz, Karin U. Löffler

**Affiliations:** 1 Sektion Ophthalmopathologie, Klinik für Augenheilkunde, Universitätsklinikum Bonn, Ernst-Abbe-Str. 2, 53127 Bonn, Deutschland; 2 Klinik für Augenheilkunde, Universitätsklinikum Bonn, Bonn, Deutschland

Wir berichten über 2 Patientinnen, die sich aufgrund eines ungewöhnlichen Bindehauttumors, der erst im Erwachsenenalter auftrat, in unserer Ambulanz vorstellten. Die endgültige Diagnose konnte in beiden Fällen erst nach der histologischen Untersuchung gesichert werden.

## Patientin 1

### Anamnese

Eine 65-jährige Patientin stellte sich erstmalig zur Mitbeurteilung einer seit 8 Jahren bestehenden Bindehautveränderung am rechten Auge in unserer Sprechstunde vor. Dem niedergelassenen Augenarzt sei im letzten Jahr eine Größenveränderung aufgefallen.

Die Patientin gab an, dass ihr die Veränderung keine Beschwerden bereite. Anamnestisch berichtete die Patientin von einer Augenverletzung durch einen Feuerwerkskörper am rechten Auge als Vierjährige. Seitdem seien am temporalen Ober- und Unterlid keine Wimpern mehr gewachsen. Bei den damaligen Kontrollen durch den Augenarzt nach der Verletzung sei die Bindehautläsion noch nicht vorhanden gewesen. An Allgemeinerkrankungen lagen ein Diabetes mellitus Typ 2 sowie eine Hypothyreose vor. Einen Monat zuvor sei ein Herzschrittmacher implantiert worden.

### Befunde

**Figure 1:**
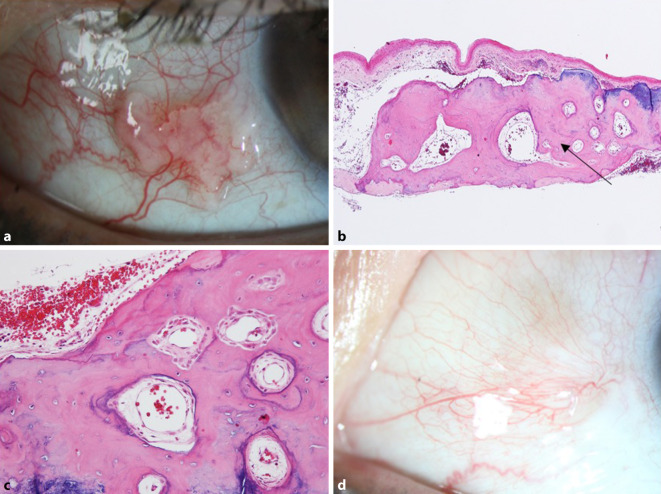

Der bestkorrigierte Visus betrug bei Erstvorstellung kataraktbedingt 0,5 beidseits. Spaltlampenmikroskopisch zeigte sich beidseits eine Katarakt bei ansonsten reizfreiem vorderem Augenabschnitt. Am rechten Auge zeigte sich temporal ein gut verschieblicher weißlich-granulärer Bindehauttumor mit glänzender Oberfläche (Abb. 1a). Unter der Verdachtsdiagnose einer atypischen Pinguecula, aber letztendlich noch unklarer Diagnose, wurde die Bindehautveränderung in toto exzidiert und histopathologisch untersucht. Beim Zuschneiden des Präparates erwies sich das Präparat (8 × 7 × 1,5 mm) als extrem hart. Zur Optimierung der Schnittqualität wurde das Gewebestück entkalkt. Mikroskopisch zeigte sich am Ober- und Unterrand becherzellarmes, nicht verhornendes Plattenepithel ohne signifikante Atypien über einer deutlichen elastotischen Degeneration des darunter gelegenen Bindegewebes. Der zentrale Anschnitt zeigte hingegen nicht verhornendes Plattenepithel mit einer darunterliegenden Schicht aus verdichtetem Bindegewebe, das morphologisch dem Epithel der Kornea mit darunterliegender Bowman-Schicht ähnelte. Darunter fand sich ein von Bindegewebe umgebenes Gewebe, das histologisch mit Knochengewebe vereinbar war (Abb. 1b ,c). Entzündliche Veränderungen oder Hinweise auf Malignität ergaben sich nicht.

### Diagnose

Nach der histologischen Aufarbeitung konnte die Diagnose eines epibulbären ossären Choristoms gestellt werden.

### Verlauf

Wir sahen die Patientin 10 Tage postoperativ erneut in unserer Sprechstunde. Hier zeigte sich ein regelrechter postoperativer Befund. Bei der Verlaufskontrolle 10 Monate später fand sich ein unauffälliger Befund mit kaum sichtbarer Narbe und ohne Anhalt für ein Rezidiv (Abb. 1d).

## Patientin 2

### Anamnese

Der behandelnde Augenarzt überwies eine 76-jährige Patientin mit einer neu aufgetretenen Bindehautveränderung am linken Auge. Die Veränderung war erstmals vor 1 Woche aufgefallen. In der Augenanamnese berichtete die Patientin, dass vor 18 Monaten eine seborrhoische Keratose am linken Unterlid entfernt worden sei. Zudem sei sie beidseits aufgrund peripherer Netzhautforamen gelasert worden. An allgemeinen Vorerkrankungen bestanden eine koronare Herzerkrankung sowie eine arterielle Hypertonie.

### Befunde

**Figure 2:**
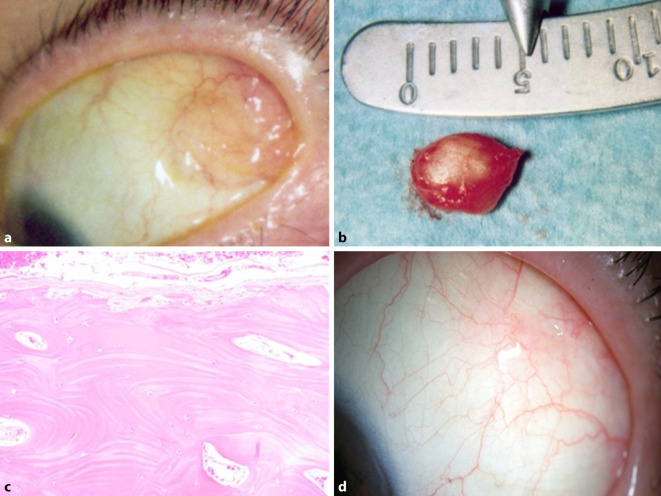

Bei der ophthalmologischen Untersuchung sahen wir am linken Auge eine weißlich-gelbliche subkonjunktivale Veränderung im temporalen oberen Quadranten (Abb. 2a) bei ansonsten reizfreier Pseudophakie. Der Durchmesser der Bindehautläsion betrug ca. 6 mm. Die Veränderung war scharf begrenzt und in ihrer Konsistenz eher hart. Da sich eine leichte Adhärenz zur Sklera zeigte, führten wir zudem eine Ultraschalluntersuchung durch. Hier zeigte sich die Läsion echoreich mit Schallauslöschung. Wir entschieden uns zur Exzision der Läsion mit anschließender histologischer Untersuchung. Intraoperativ zeigte sich die Bindehautveränderung an der Tenonschicht anhaftend, ließ sich jedoch gut mobilisieren und entfernen. Makroskopisch zeigte sich ein 7 × 5 × 1,5 mm messendes Präparat von extrem harter Konsistenz (Abb. 2b). Auch hier erfolgte eine Entkalkung vor der weiteren Aufarbeitung. Histologisch sahen wir ein von Bindegewebe umgebenes reifes Knochenstück mit Osteozyten, das von Gefäßen durchzogen war. Es zeigten sich keine Atypien oder Malignitätszeichen (Abb. 2c).

### Diagnose

Nach der histologischen Aufarbeitung konnte auch hier die Diagnose eines epibulbären ossären Choristoms gestellt werden.

### Verlauf

Wir sahen die Patientin 10 Tage sowie 3 Monate nach der Operation (Abb. 2d). Es zeigte sich bei beiden Untersuchungen kein Anhalt für ein Rezidiv.

## Diskussion

Das epibulbäre ossäre Choristom (epibulbäre Osteom) ist als normales adultes Knochengewebe in abnormaler Lokalisation definiert. Es handelt sich um eine kongenitale Neubildung mit – wenn überhaupt – langsamem Wachstum, die häufig schon im Kindesalter auftritt. Die Entstehungsursache ist nach wie vor nicht geklärt [4].

Die Erstbeschreibung erfolgte 1863 durch von Graefe [8]. Es ist insbesondere am Auge eine sehr seltene Neubildung. In der Literatur wurden bisher lediglich 65 Fälle beschrieben [2]. Abgesehen von der Manifestation am Auge können ossäre Choristome auch im Nasen-Rachen-Bereich auftreten [1]. Eine maligne Entartung wurde bisher nicht beschrieben [3].

Das Knochengewebe ist typischerweise von Bindegewebe umgeben und am häufigsten episkleral superior-temporal lokalisiert. Größere ossäre Choristome können aufgrund ihrer Ausdehnung zu Schmerzen oder einem Astigmatismus führen [7]. Histologisch zeigt sich reifes Knochengewebe mit häufig gut ausgebildeten Havers-Kanälen, welches von Bindegewebe umgeben ist.

Bei der klinischen Untersuchung kann differenzialdiagnostisch auch ein Dermoid in Erwägung gezogen werden, hier findet sich histologisch jedoch pathognomonisch vergröbertes Bindegewebe. Auch das Dermoid ist eine kongenitale Veränderung und wird häufig im Kindesalter oder frühen Erwachsenenalter diagnostiziert [6].

Bei beiden Patientinnen ist v. a. die späte Erstmanifestation ungewöhnlich. In der Literatur wird eine Entstehung ossärer Choristome auch nach Trauma diskutiert, dies wäre in den hier beschriebenen Fällen als mögliche Entstehungsursache in Betracht zu ziehen [5]. Aufgrund der Lokalisation und der histologisch sichtbaren elastotischen Degeneration ist bei der ersten Patientin möglicherweise auch an eine Ossifikation einer Pinguecula zu denken. Hierzu findet sich nach aktueller Recherche keine weitere Beschreibung in der Literatur.

## Fazit für die Praxis

Epibulbäre ossäre Choristome sind als adultes Knochengewebe in abnormaler Lokalisation definiert und eine seltene Neubildung, die meist im Kindes- oder frühen Erwachsenenalter diagnostiziert wird. Eine späte Manifestation wie in unserer Kasuistik ist untypisch. Letztendlich ist hier die histologische Aufarbeitung essenziell für die Diagnosestellung.
